# Raster-scanning serial protein crystallography using micro- and nano-focused synchrotron beams

**DOI:** 10.1107/S1399004715004514

**Published:** 2015-04-25

**Authors:** Nicolas Coquelle, Aaron S. Brewster, Ulrike Kapp, Anastasya Shilova, Britta Weinhausen, Manfred Burghammer, Jacques-Philippe Colletier

**Affiliations:** a Université Grenoble Alpes, IBS, 38044 Grenoble, France; b CNRS, IBS, 38044 Grenoble, France; c CEA, IBS, 38044 Grenoble, France; d Physical Biosciences Division, Lawrence Berkeley National Laboratory, Berkeley, CA 94720, USA; e European Synchrotron Radiation Facility, BP 220, 38043 Grenoble, France; f Department of Analytical Chemistry, Ghent University, Ghent B-9000, Belgium

**Keywords:** serial crystallography, hit finding, raster scanning, background subtraction, radiation damage

## Abstract

High-resolution structural information was obtained from lysozyme microcrystals (20 µm in the largest dimension) using raster-scanning serial protein crystallography on micro- and nano-focused beamlines at the ESRF. Data were collected at room temperature (RT) from crystals sandwiched between two silicon nitride wafers, thereby preventing their drying, while limiting background scattering and sample consumption. In order to identify crystal hits, new multi-processing and GUI-driven Python-based pre-analysis software was developed, named *NanoPeakCell*, that was able to read data from a variety of crystallographic image formats. Further data processing was carried out using *CrystFEL*, and the resultant structures were refined to 1.7 Å resolution. The data demonstrate the feasibility of RT raster-scanning serial micro- and nano-protein crystallography at synchrotrons and validate it as an alternative approach for the collection of high-resolution structural data from micro-sized crystals. Advantages of the proposed approach are its thriftiness, its handling-free nature, the reduced amount of sample required, the adjustable hit rate, the high indexing rate and the minimization of background scattering.

## Abstract 2

A raster scanning serial protein crystallography approach is presented, that consumes as low ∼200–700 nl of sedimented crystals. New serial data pre-analysis software, *NanoPeakCell*, is introduced.

## 1. Introduction   

The advent of X-ray free-electron lasers (XFELs) has driven the emergence of serial protein crystallography (Chapman *et al.*, 2011 ▶). In this novel approach, data are collected at room temperature (RT) from tens to hundreds of thousands of ‘still’ crystals, all of which are in different, more or less random, orientations. In contrast, the vast majority of the nearly 94 000 X-ray structures deposited in the PDB were obtained from data collected at cryogenic temperature (generally 100 K) from a single crystal rotated in an X-ray beam. Compared with RT, such low temperatures reduce by ∼100-fold the radiation damage that accumulates in crystals during data collection and degrades the structural information (Nave & Garman, 2005 ▶). The price to pay is, however, that of reduced insight into protein conformational heterogeneity and dynamics (Motlagh *et al.*, 2014 ▶; Fraser *et al.*, 2011 ▶). Kinetic crystallography approaches have been developed to overcome the static nature of cryo-crystallographic structures, but none are universally applicable (Bourgeois & Royant, 2005 ▶). Arguably, methodologies to collect high-resolution X-ray data at RT are needed to better understand the interplay between structure and dynamics and how they synergistically give rise to protein function (Weik & Colletier, 2010 ▶).

XFELs deliver roughly as many photons in a 5–50 fs pulse as an insertion-device beamline at a third-generation synchrotron does in a second. Such short exposures allow the collection of RT diffraction data before crystals are destroyed by the intense X-ray pulse (Chapman *et al.*, 2011 ▶; Neutze *et al.*, 2000 ▶). At XFELs, samples are commonly streamed across the X-ray beam by virtue of an injector. Because crystals virtually stand still (no oscillation or tumbling) during the time-lapse of their exposure to the X-rays, only a thin slice of the reciprocal space is recorded for each. The production of a full data set therefore requires the collection of patterns from many thousands of crystals in an approach termed ‘serial femtosecond crystallography’ (SFX; Chapman *et al.*, 2011 ▶). Over the last four years, this approach has demonstrated its power, notably by enabling high-resolution data to be collected from submicrometre-sized crystals (Boutet *et al.*, 2012 ▶; Koopmann *et al.*, 2012 ▶; Redecke *et al.*, 2013 ▶; Sawaya *et al.*, 2014 ▶; Stevenson *et al.*, 2014 ▶). XFELs also hold the promise of soon allowing subpicosecond time-resolved structural studies, which are technically unfeasible at third-generation synchrotron radiation (SR) sources (Neutze, 2014 ▶). These SFX developments have been accompanied by active software development in order to contend both with the thinness of the three-dimensional reciprocal slice collected from still crystals and with the colossal amount of data produced during experiments.

In SFX, samples are often presented to the X-ray beam by means of gas dynamic virtual nozzle (GDVN)-type injectors. GDVNs can be used to compress an aqueous crystal stream, thereby generating a liquid jet (DePonte *et al.*, 2009 ▶). They can also be used to push a crystal-containing lipid cubic phase (LCP) across the X-ray beam (Weierstall *et al.*, 2014 ▶). Liquid GDVN injectors require monumental amounts of sample (hundreds of milligrams), and thus are only suited for targets whose production, purification and crystallization are straightforward and streamlined. LCP injectors are slower and therefore comparatively economical (<1 mg), but the range of protein crystals that are amenable to embedding in LCP is as yet unknown. Also, resuspending crystals into LCP can be difficult and LCP injectors can suffer from drying and phase transitions. A possible alternative is to embed crystals into mineral grease and to use an LCP injector to flow them across the X-ray beam (Sugahara *et al.*, 2015 ▶). A nanoflow electro-spinning injector has been described in which high voltages are used instead of high pressures to flow crystals across the X-ray beam (Sierra *et al.*, 2012 ▶). Use of this technology reduces the amount of sample required down to hundreds of micrograms (Sierra *et al.*, 2012 ▶), but requires that crystals are grown in, or are transferable into, a viscous mother liquor. A system based on acoustic droplet ejection has also been described (Soares *et al.*, 2011 ▶). Common to all these approaches is that crystals are literally drowned in solvent, leading to high and fluctuating background scattering that complicates data processing. Alternate sample-handling methods are therefore amongst the most critical developments required for the further emergence of SFX. Recently, a method for the collection of data from crystalline samples dried onto fixed targets has been presented (Hunter *et al.*, 2014 ▶), as well as a goniometer-based approach to SFX (Cohen *et al.*, 2014 ▶).

The worldwide availability of SR sources raises the possibility that some of the experiments currently performed at XFELs could be implemented at synchrotrons. As already mentioned, the excitement elicited by XFELs has led scientific programmers to challenge themselves with the handling and processing of millions of diffractions patterns, making it now possible to process serial crystallographic data regardless of the X-ray sources they are collected at. To date, at least two major software suites exist: *CrystFEL* (White *et al.*, 2012 ▶) and *cctbx.xfel* (Sauter *et al.*, 2013 ▶). Subtleties in the processing approaches of the two software suites have recently been discussed (Sawaya *et al.*, 2014 ▶).

In early 2014, a pioneering study reported serial synchrotron crystallography (SSX) experiments on *Trypanosoma brucei* cathepsin B crystals (CatB) mounted in a nylon loop at 110 K (Gati *et al.*, 2014 ▶). Data were collected using a combination of raster scanning and oscillation, whereby evenly spaced (5 µm) volumes of the nylon-loop content were illuminated by a 4 × 5 µm beam while oscillating the loop by 90° over the complete scan. Hence, a three-dimensional wedge, rather than a thin slice of the reciprocal lattice, was collected at each exposure. More recently, another consortium reported serial synchrotron crystallography data collected at RT from lysozyme crystals flowed across a 6 × 9 µm X-ray beam inside a capillary (Stellato *et al.*, 2014 ▶). Using this new approach, the authors were able to collect ∼1.6 × 10^6^ frames in about 17 h, of which ∼40 × 10^3^ were successfully indexed. A total of 2.5 ml crystal slurry was consumed, corresponding to 250 mg of protein. The data proved to be of excellent quality [as judged from statistical indicators such as *R*_split_, 〈*I*/σ(*I*)〉, CC_1/2_ and redundancy], producing a 2.1 Å resolution structure comparable to that obtained by the collection of oscillation data at a cryogenic temperature. Two compelling advantages of this approach are that (i) pseudo-oscillation data can be collected from crystals rolling inside the capillary during the exposure time and (ii) frames can be acquired in a shutterless fashion, thus theoretically reducing the exposure time to the limit of detector readout. Among the remaining issues are, however, the amount of sample consumed and the overall success rate. A more economical approach is thus still required for proteins whose production and crystallization cannot reach a manufacturing scale.

In the present study, we report an alternative serial synchrotron crystallography approach allowing the collection of data at RT and in a pure raster-scanning mode; that is, without oscillating the crystals. For this proof of concept, we used tetragonal microcrystals of chicken hen egg-white lysozyme so as to allow a direct comparison of our data with those recently produced by SFX (Boutet *et al.*, 2012 ▶) and SSX (Stellato *et al.*, 2014 ▶). To determine the minimal beam size to perform such experiments at SR facilities, data were collected using either a micro-focused or a nano-focused beam. Samples were presented to the X-ray beam in a silicon nitride (Si_3_N_4_) sandwich, thence avoiding the drying of samples while limiting background scattering and sample consumption. Identification of crystal hits, background subtraction and peak searching were performed using *NanoPeakCell*, a new multi-processing and GUI-driven Python-based pre-analysis software. Further processing of the data was carried out with *CrystFEL*, and structures collected using the micro- and nano-focused beams were refined to 1.7 Å resolution. We propose the use of RT raster-scanning serial protein crystallography using SR micro- and nano-focused beams as an alternative approach for the collection of high-resolution structural data from micrometre-sized crystals. The proposed method is thrifty and virtually handling-free, and so can be used on fragile and scarce microcrystalline samples.

## 2. Materials and methods   

### 2.1. Description of the ESRF Microfocus Beamline (ID13)   

ID13 is a versatile beamline that provides ESRF users with intense micro-focused (0.3–2.5 µm, micro-branch, EH2) and nano-focused (100–500 nm; nano-branch, EH3) beams. The focusing optics used to generate the micro-beam are Be-based compound refractive lenses (CRL) consisting of 56 single lenses. Each lens has a curvature radius of 50 µm in the apex. For the nano-beam, Si-based linear compound refractive lenses in crossed geometry (so-called NFL lenses; Schroer *et al.*, 2005 ▶) are used.

At the micro-branch of ID13, sample centring and positioning are performed with translation stages (Micos, Germany), with 200 nm incremental steps in the *x*/*y* directions (HPS-170) and an overall travel range of 160 mm, and incremental steps of 100 nm in the vertical direction (UPL-160) with a travel range of 25 mm. In the nano-branch, the sample centring and scanning are operated *via* a P622K075 piezo *z* stage (vertical direction) mounted on a P622K074 piezo *xy* stage. The minimum incremental movement as mounted on the nano-setup of ID13 is 10 nm in the vertical direction and 20 nm in the two horizontal directions. This arrangement of piezo stages is mounted on an M-810 miniature hexapod. The piezo stages and the hexapod were manufactured by Physik-Instrumente (PI GmbH, Karlsruhe, Germany). The hexapod and the piezo stage can translate in the three spatial dimensions over distances of about 10 mm and 250 µm, respectively.

Diffraction data were recorded using an ESRF-built Frelon 4M CCD area detector with 2048 × 2048 pixels and 50 µm pixel size (Coan *et al.*, 2006 ▶) in the 2× binning mode (1024 × 1024 pixels and 100 µm pixel size; ∼0.35 s readout time). Data were collected in raster scanning mode, i.e. by translating samples through the focus in two directions perpendicular to the incident beam.

### 2.2. Sample preparation and data collection   

Tetragonal (*P*4_3_2_1_2) micro-crystals (20 × 20 × 20 µm) of hen egg-white lysozyme (HEWL) were grown using the vapour-diffusion method (Lomb *et al.*, 2011 ▶) and directly pipetted from their drops into an Eppendorf tube. Gentle centrifugation for 300 s (6000 rev min^−1^) settled the crystals at the bottom of the tube.

**Figure 1:**
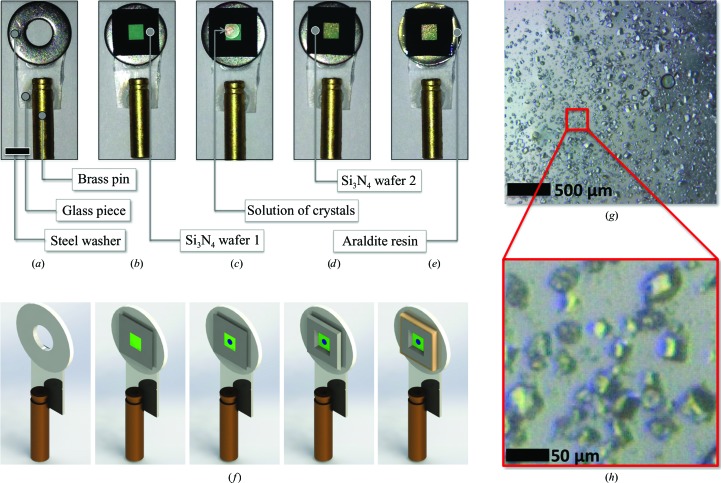
Sample presentation to the X-ray beam. (*a*) A brass pin and a washer are glued onto a piece of glass. (*b*) The concave surface of the first Si_3_N_4_ wafer is glued onto the washer. (*c*) 500 nl of crystalline slurry is gently deposited onto the membrane. (*d*) The second Si_3_N_4_ wafer is sandwiched over the first. (*e*) The sandwich is sealed with Araldite resin in order to avoid drying of the material. Following this step, the brass pin is introduced into a magnetic crystal mount. (*f*) Three-dimensional rendering of steps (*a*)–(*e*). (*g*, *h*) Overview (*g*) and close-up view (*h*) of the lysozyme crystals in the Si_3_N_4_ sandwich.

500 nl of the crystalline precipitate were pipetted onto a 500 nm thick Si_3_N_4_ wafer (frame thickness 200 µm) before laying another Si_3_N_4_ wafer (of the same thickness) onto it in a back-to-back fashion (Fig. 1 ▶). Si_3_N_4_ wafers were from Silson, Northampton, England (http://www.silson.com). Sandwiching the Si_3_N_4_ wafers back to back was essential to prevent crystals from slithering down during the diffraction experiment. The Si_3_N_4_ sandwich was placed over a steel washer, glued onto it using SuperGlue and the sandwich border was hermetized using Araldite resin (Fig. 1 ▶ *a*). The washer was glued onto a small glass piece, itself glued onto a brass pin that was introduced into an adjustable magnetic crystal mount. The usable Si_3_N_4_ surface was 2.5 × 2.5 mm. For presentation to the X-ray beam, the mounts were either placed onto an *xyz* translation table (micro-focused beam; ESRF ID13-EH2) or onto a miniature hexapod (nano-focused beam; ESRF ID13-EH3). Data sets collected using the micro- and nano-focused beams are referred to as ‘micro’ and ‘nano’, respectively. Data collection was segmented into scans of 41 (horizontal, fast scanning) by 41 (vertical, slow scanning) steps, each started at a remote location on the wafer. The track length of photoelectrons from 1 Å wavelength X-rays in water and protein has been estimated to be ∼3 µm (Cole, 1969 ▶). Within the same scan, X-ray shots were accordingly spaced by 10 (>2.5 + 3) and 5 (>0.2 + 3) µm for the micro (beam size 1.5 × 2.5 µm) and nano (beam size 0.15 × 0.18 µm) data sets, respectively (Table 1 ▶).

Exposure times were 0.2 and 0.1 s per shot for the micro and nano data sets, respectively. The flux densities of the micro- and nano-focused beams were 2.67 × 10^16^ and 6.48 × 10^17^ photons s^−1^ mm^−2^, respectively, while the calculated doses per image (and thus the average doses of the data set) were 3.2 and 29.1 MGy, respectively (Table 1 ▶). The micro and nano data sets consist of ∼41 and ∼84 scans (69 319 and 139 985 frames), corresponding to ∼9 and 20 h of data collection, respectively. The rate of data acquisition was limited by the readout time of the detector.

### 2.3. Data sorting and processing   

**Figure 2:**
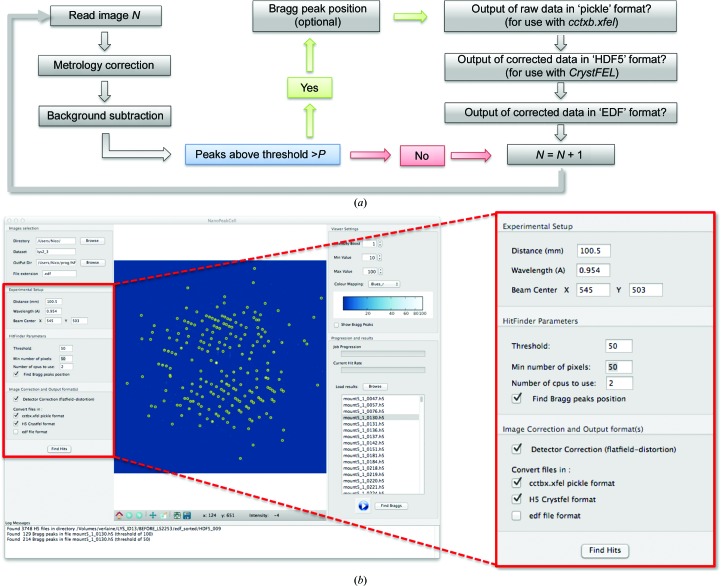
The new *NanoPeakCell* pre-analysis software for serial crystallography. (*a*) Workflow of the actions performed by *NanoPeakCell*. (*b*) Overview of the graphical user interface (GUI) of *NanoPeakCell*. On the left, the user can define important experimental parameters, as well as parameter values used during the hit-finding and peak-finding procedures (red inset). On the bottom left, the user can select the correction(s) to be performed and the output format(s) for saving hits (red inset). Each hit is displayed in the central panel, with yellow circles around Bragg peaks. *NanoPeakCell* can also be used as a data-visualization program. The right panel is dedicated to display adjustments and allows results from previous runs of *NanoPeakCell* to be reloaded for inspection. Users can also use *NanoPeakCell* to view raw data in any of the supported formats.

**Figure 3:**
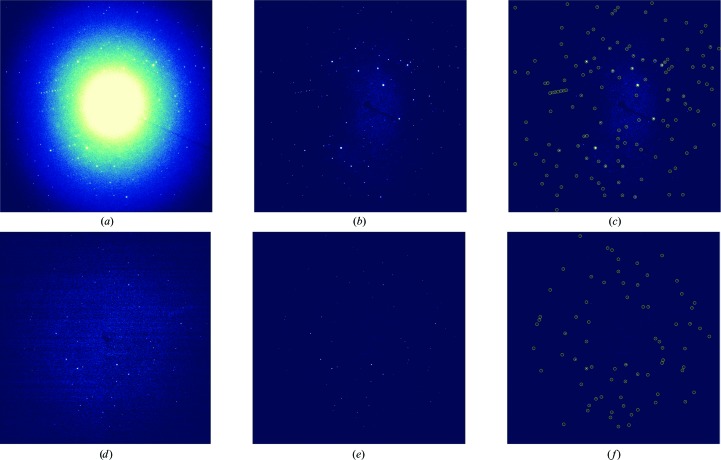
Data correction and finding Bragg peaks. (*a*), (*b*) and (*c*) show the same diffraction pattern from the micro data set before (*a*) and after (*b*, *c*) background subtraction. (*c*) shows the Bragg peaks found by *NanoPeakCell* in this diffraction pattern. Likewise, (*d*), (*e*) and (*f*) show the same diffraction pattern from the nano data set before (*d*) and after (*e*, *f*) background subtraction. The Bragg peaks found by *NanoPeakCell* are highlighted in (*f*) (yellow circles).

Regardless of whether the data are collected at an XFEL or at an SR source, a major challenge in serial crystallography is hit finding. Also, background scattering must be subtracted to permit an accurate measurement of the diffracted intensities (Boutet *et al.*, 2012 ▶). Data correction must, however, be performed cautiously. Indeed, in a given volume crystals and solvent are mutually exclusive, so the amount of background to be subtracted needs to be scaled on a per-pattern basis. Specific to the use of a Frelon CCD camera, raw images must also be corrected for distortion and a flat-field correction applied (metrology correction). Together, these requirements prompted us to undertake the coding of a multi-processing, GUI-driven Python-based hit finder and corrector, which we named *NanoPeakCell*. The flowchart of actions performed by *NanoPeakCell* is depicted in Fig. 2 ▶(*a*) and includes conversion of images (the supported input formats include EDF, SMV, MCCD, CBF, TIFF, HDF5, SACLA-HDF5 and LCLS-XTC) into a suitable format for data processing with *CrystFEL* (HDF5 format) and/or *cctbx.xfel* (‘pickle’ format), as well as the generation of the parameter files and scripts needed to run *CrystFEL* in a user-friendly fashion (Fig. 2 ▶ *b*). Of note, *NanoPeakCell* not only selects hits (*i.e* frames with actual diffraction spots) on the basis of a single pixel being higher than a given threshold, but also checks whether a sufficient (user-provided) number of such pixels are present to allow further indexing. To allow determination of the effective resolution limit of the serial data set, *NanoPeakCell* additionally performs a maximum projection of selected hits on the fly (see Supplementary Fig. S1). Finally, *NanoPeakCell* performs Bragg-peak finding using a local maximum algorithm and outputs the peak list in a supplementary data set of the HDF5 file, if the user selects for this output format, or in a text file otherwise. This can allow the rapid elimination of both blank and low-resolution images. Similar indexing rates were obtained when using the peak-finding function implemented in *CrystFEL* and that implemented in *NanoPeakCell*. Using the latter, however, avoids spending time optimizing the peak-detection parameters in *CrystFEL*. Accordingly, in this work, Bragg-peak finding was performed using *NanoPeakCell* and peak locations were fed into *CrystFEL* (using the --­peaks=hdf5 option in the *indexamajig* module) for indexing (Kirian *et al.*, 2011 ▶; White *et al.*, 2012 ▶, 2013 ▶). *NanoPeakCell* is installed on the ESRF ID13 micro-focused (ID13-EH2) and nano-focused beamlines (ID13-EH3), where it can be controlled either through the GUI or from the command line, and its development is continuing. Typical raw and corrected images from the micro and nano data sets are shown in Fig. 3 ▶. Others details concerning *NanoPeakCell* and its use at various facilities (including ESRF, LCLS and SACLA) will be published elsewhere.

Data were processed using *CrystFEL* (Kirian *et al.*, 2011 ▶; White *et al.*, 2012 ▶, 2013 ▶). *CrystFEL* indexing relied on *MOSFLM* (Powell *et al.*, 2013 ▶), *Dirax* (Duisenberg, 1992 ▶) and *XDS* (Kabsch, 2010 ▶). Bragg-peak integration was performed using the ‘rings-nocen’ method in *CrystFEL*, with concentric rings of five, seven and nine pixels to determine the peak, buffer and background regions, respectively. The radius of the three-dimensional intensity profile was 5 × 10^−4^ Å^−1^. The divergences of the micro- and nano-focused beams were determined experimentally (Table 1 ▶). Merging of integrated intensities was performed using Monte Carlo integration in *CrystFEL* (Kirian *et al.*, 2011 ▶; Table 2 ▶).

The initial data processing was performed using conservative 〈*I*/σ(*I*)〉 (>2) and *R*_split_ (∼50%) criteria to determine the highest resolution shells. Based on these criteria, the micro and nano data sets were integrated to 1.95 and 1.85 Å resolution, respectively (Table 2 ▶). These two data sets, and their corresponding structures, are referred to as ‘conservative micro’ and ‘conservative nano’. The statistics of the conservative data sets, shown in Table 2 ▶, are of excellent quality, as judged by *R*_split_, CC_1/2_ and Wilson *B* factor. Supplementary Fig. S2 shows the convergence of data-quality indicators such as *R*_split_ and CC_1/2_ as a function of resolution and the number of images. It indicates that in both data sets the CC_1/2_ values at 1.7 Å resolution remain well above the 0.135 limit advised by Karplus & Diederichs (2012 ▶). The presence of diffraction signal up to this resolution in both data sets is also visible in the azimuthal integrations of the maximum projections of the indexed patterns (Supplementary Fig. S1). Thus, we performed a more progressive integration, in which CC_1/2_ and iterative pair refinement (Karplus & Diederichs, 2012 ▶) were used instead of strict 〈*I*/σ(*I*)〉 and *R*_split_ criteria to determine the high-resolution cutoffs. These two 1.7 Å resolution data sets and their corresponding structures are referred to as ‘progressive micro’ and ‘progressive nano’. The only difference between the progressive nano (or progressive micro) and the conservative nano (or conservative micro) data sets is thus the resolution cutoff.

In order to determine whether it is the greater number of images or the smaller beam size that explain the much better statistics of the nano data sets (both conservative and progressive) when compared with those of the micro data sets, we also generated ‘reduced nano’ data sets by randomly selecting, in the nano data sets, a number of hits equivalent to that used to produce the micro data set. The same number of indexed patterns was thus used to produce the progressive reduced nano and the conservative reduced nano data sets, which only differ in the resolution cutoff.

### 2.4. Structure determination and analysis   

Data were converted to mtz format and phased by molecular replacement using *Phaser* (McCoy *et al.*, 2007 ▶) with PDB entry 1ljn (Harata & Kenai, 2002 ▶) as the starting model. To verify whether the structural information contained in our data sets could have allowed automated model building, we attempted fitting of the full HEWL amino-acid sequence into various molecular-replacement maps using *phenix.autobuild* with default settings (Terwilliger *et al.*, 2008 ▶). The maps were generated from molecular-replacement trials with six different lysozyme models. The first three models consisted of the main-chain and side-chain atoms from residues 1–127 (full protein), 1–65 (N-terminal half) or 66–127 (C-terminal half). The other three were composed of the main-chain atoms of residues 1–127, 1–65 or 66–127. In all cases, ∼98% of the main chain and 90–98% of the side chains were reconstructed (Supplementary Table S1).

**Figure 4:**
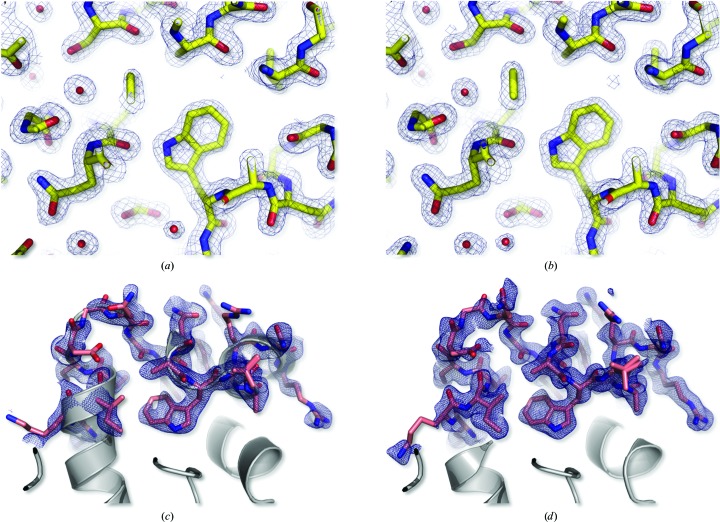
Unbiased 2*mF*_o_ − *DF*_c_ OMIT electron-density maps. (*a*, *b*) Overview of the electron density around Trp108 in unbiased 2*mF*_o_ − *DF*_c_ OMIT electron-density maps (contour level 1.2σ) calculated from the progressive micro (*a*) and progressive nano (*b*) data sets. (*c*, *d*) Unbiased 2*mF*_o_ − *DF*_c_ electron-density maps (contour level 1.2σ) calculated from the micro (*c*) and nano (*d*) data sets using as a starting model a truncated model of lysozyme lacking residues 96–116. Residues 96–116 are shown as pink sticks, while the residues used for generating the maps are shown as ribbon diagrams. That the electron-density maps shown in (*c*) and (*d*) cover virtually all of the atoms in residues 96–116 highlights the quality of the structural information contained in the progressive micro and progressive nano data sets.

Structures were refined by iterative cycles of reciprocal-space and real-space refinement. Reciprocal-space refinement was performed using the *PHENIX* software suite (Adams *et al.*, 2010 ▶) and included refinement of coordinates and atomic displacement parameters (*B* factors). Manual modifications in real space were performed using *Coot* (Emsley & Cowtan, 2004 ▶). Unbiased refined 2*mF*_o_ − *DF*_c_ and *mF*_o_ − *DF*_c_ electron-density maps are shown in Figs. 4 ▶(*a*) and 4 ▶(*b*) for the two progressive data sets. To further assess the quality of these data, residues 96–116 were deleted from the model and reciprocal-space refinement was undertaken (eight cycles of positional and *B*-factor refinement, including two cycles of simulated annealing). In the resulting 2*mF*_o_ − *DF*_c_ map from both data sets, quasi-perfect electron density is observed around the omitted residues (Figs. 4 ▶ *c* and 4 ▶ *d*).

We attempted to compare our data sets (conservative micro and conservative nano) with a variety of previously published HEWL data sets, including (i) two low-dose data sets collected using conventional oscillation methods either at 100 K (PDB entry 1vds; S. Aibara, A. Suzuki, A. Kidera, K. Shibata, T. Yamane, L. J. DeLucas & M. Hirose, unpublished work; data collected on a rotating anode) or RT (PDB entry 3zek; *in situ* data collection on a synchrotron beamline; Pinker *et al.*, 2013 ▶), (ii) an SFX data set collected in vacuum and at RT using 40 fs XFEL pulses (PDB entry 4et8; Boutet *et al.*, 2012 ▶) and (iii) an SSX data set collected at RT by flowing a crystal slurry across a synchrotron X-ray beam (PDB entry 4o34; Stellato *et al.*, 2014 ▶). In the following, these data sets (PDB entries 1vds, 3zek, 4et8 and 4o34) are referred to as ‘low-dose 100 K’, ‘low-dose 295 K’, ‘SFX’ and ‘Flow-SSX’, respectively. Data sets were scaled using *SCALEIT* from *CCP*4 (Winn *et al.*, 2011 ▶; Supplementary Figs. S3 and S4). The unweighted *R*_iso_ values between the various data sets are high, suggesting non-isomorphism, whereas the compilation of unit-cell parameters rather points towards all data sets (and the resulting structures) being isomorphous. A possible hypothesis to explain this apparent lack of isomorphism is that it emerges from errors in intensity distributions introduced by the Monte Carlo integration. Accordingly, and as observed previously by Schlichting and coworkers, the weighted *R* factors are drastically lower (Boutet *et al.*, 2012 ▶), in particular at low resolution. However, weighted *R* factors may not be the most appropriate metric to directly compare data sets collected using different methods, owing to multiple systematic errors in signal-to-noise estimation and scaling. Regardless, our data tend to better agree with the data collected using conventional methods than with the data collected using Flow-SSX or SFX. A possible explanation is that errors introduced during the Monte Carlo integration of the various serial data sets add up during the scaling procedure.

Structure-factor amplitude Fourier difference (*F*_o_ − *F*_o_) maps were calculated between the various data sets after the structure-factor amplitude differences were *q*-weighted to meliorate their estimates (Colletier *et al.*, 2007 ▶). *F*_o_ − *F*_o_ maps between data sets *i* and *j* were thus computed using observed structure-factor amplitudes of data sets *i* and *j* and calculated phases from model *i*, *i.e.* [*qw*(*F*_o_ *^j^* − *F*_o_ *^i^*)]exp(−*i*ϕ^*j*^). For comparison purposes, all *F*_o_ − *F*_o_ maps were computed to a resolution of 2.1 Å (*i.e.* the resolution of the Flow-SSX data set). The figures were produced using *PyMOL* (Schrödinger), *gnuplot* or home-made Python scripts.

## 3. Results   

### 3.1. Data processing   

We used *NanoPeakCell* (Fig. 2 ▶) to identify hits, correct the metrology, subtract background scattering and locate Bragg peaks (Fig. 3 ▶). The hit rates were 9.9 and 41.9% for the micro and nano data sets, respectively (*i.e.* 6862 and 58 647 frames, respectively; Table 2 ▶). The drastically lower hit rate attained for the micro data set can be partly explained by the reduced probability of hitting the same crystal twice when the step size of the raster scan is doubled. It is, however, more likely that the crystal concentration in the Si_3_N_4_ sandwich used to collect the micro data set was sub-optimal.

Indexing and integration were performed using *CrystFEL*. Data-processing parameters, as well as subtleties specific to serial synchrotron data-processing, are detailed in §[Sec sec2]2. Briefly, 85 and 80% of *NanoPeakCell* hits were indexed and integrated for the micro and nano data sets, respectively (Table 2 ▶). Reaching such high indexing rates was only possible with background-subtracted data. Merging was performed by Monte Carlo integration (Kirian *et al.*, 2011 ▶). For each data set, two integrations were performed using either conservative (1.95 and 1.85 Å resolution cutoffs for the conservative micro and conservative nano data sets, respectively) or progressive (1.7 Å resolution cutoffs for the progressive micro and progressive nano data sets) criteria.

### 3.2. Quality of the structural information   

All data sets were phased by molecular replacement (MR) using the structure of HEWL in the tetragonal space group (PDB entry 1ljn) as the starting model. The resulting experimental maps were of excellent quality, as shown in Fig. 4 ▶ for the progressive micro and progressive nano (1.7 Å resolution) data sets. To further evaluate the quality of the structural information contained in our data sets, MR phasing was attempted using incomplete starting models. All MR trials succeeded, even when as few as 20% of the total atoms of HEWL were used. The resulting experimental maps were of sufficient quality to allow the automated building of ∼98% of the main-chain atoms and 90–98% of the side-chain atoms. Supplementary Table S1 recapitulates these results for the progressive nano data set.

The structures derived from our six data sets (conservative, progressive and progressive reduced micro and conservative, progressive and progressive reduced nano) were refined independently. The final statistics of the conservative micro and nano structures are of comparable quality (Table 2 ▶), and likewise for the progressive micro and nano structures. To decide on the effective resolution cutoffs to apply to the progressive micro and progressive nano data sets, we performed iterative pair refinement as described in Karplus & Diederichs (2012 ▶). For both progressive data sets, we observed a decrease in the *R*_free_ value at resolution *A* when the model was refined including structural information up to a higher resolution *B*. This demonstrates that in both data sets valid structural information is present beyond the conservative resolution cutoff (1.95 and 1.85 Å, respectively; Supplementary Fig. S2). The CC_1/2_ values in the highest resolution shell (1.78–1.70 Å) are 0.66 and 0.85 for the progressive micro and progressive nano data sets, respectively. The corresponding 〈*I*/σ(*I*)〉 values drop below 1 (0.64 and 0.87 for the progressive micro and progressive nano data sets, respectively) and the *R*_split_ values rise above 100%. For the sake of completeness and consistency we have deposited four models in the PDB (Table 2 ▶) corresponding to the progressive and conservative integrations of the micro and nano data sets, respectively.

### 3.3. Radiation damage   

**Figure 5:**
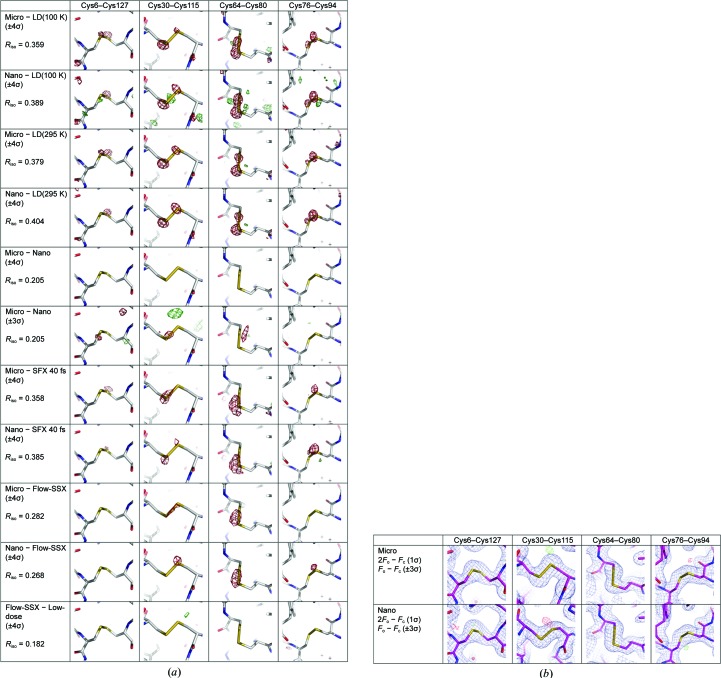
Specific radiation damage is observed in both the micro and the nano data sets, but does not compromise the structural information. (*a*) Structure-factor amplitude Fourier difference (*F*_o_ − *F*_o_) maps were calculated between our data sets (conservative micro and conservative nano) and four other HEWL data sets: the SFX data set, the Flow-SSX data set and two low-dose data sets collected at either 100 or 295 K. The maps are displayed around the four disulfide bridges of lysozyme, with red and green contours indicating negative and positive density, respectively. (*b*) Unbiased 2*mF*_o_ − *DF*_c_ (blue; contour level 1.0 σ) and *mF*_o_ − *DF*_c_ (red and green; contour level ± 3σ) maps are displayed around the four disulfide bridges of lysozyme.

It has been shown that beyond the limit of 29 MGy, the biological information obtained from a protein crystal at 100 K is compromised (Owen *et al.*, 2006 ▶). At RT, the extent of radiation damage is increased by ∼100-fold (Nave & Garman, 2005 ▶), suggesting a dose limit of 0.3 MGy. Here, the doses per diffraction pattern, and hence per data set, are 3.2 and 29.1 MGy for the micro and nano data sets, respectively. It was therefore expected that strong radiation damage would occur, notably at highly radiation-sensitive sites such as disulfide bridges. Accordingly, we observed, in structure-factor amplitude Fourier difference (*F*_o_ − *F*_o_) maps calculated between our data sets and low-dose data sets collected at both 100 K (low-dose 100 K) and RT (low-dose 295 K), strong negative peaks (−4σ) on the S atoms of HEWL, and most particularly, on disulfide bridges (Fig. 5 ▶ *a*). Interestingly, we observed that the disulfide bridge Cys64–Cys80 is the most radiation-sensitive at RT, whereas it is Cys30–Cys115 that is the most sensitive at 100 K (Sutton *et al.*, 2013 ▶). Specific damage was, however, unapparent in 2*mF*_o_ − *DF*_c_ and *mF*_o_ − *DF*_c_ maps, indicating that disulfide bridges are not broken in the micro and the nano data sets (Fig. 5 ▶ *b*). It has been shown that upon X-ray damage disulfide bridges contract (Sutton *et al.*, 2013 ▶) and/or elongate (Carpentier *et al.*, 2010 ▶; Weik *et al.*, 2002 ▶) before breaking. It may thus be that in our structures disulfide bridges are trapped in one of these intermediate stages. Regardless, Fig. 5 ▶(*b*) indicates that the extent of radiation damage is limited in our data sets and that the structural information has thus not yet been compromised.

The approximately tenfold higher flux density of the nano-focused beam was suggestive of possible higher radiation damage in the nano data set compared with the micro data set. Surprisingly, however, the *F*_o_^micro^ − *F*_o_^nano^ map did not indicate stronger damage to disulfide bridges in the nano structure. Rather, it revealed a small (−3σ) negative peak at the most sensitive disulfide bridge at RT (Cys64–Cys80), suggesting that the micro data set is more damaged than the nano data set. A possible rationale for this observation is thus that for the order-of-magnitude higher dose rate of the nano data set (291 MGy s^−1^) a lag phase occurs that delays the appearance of specific radiation damage. Additional experiments will be required in order to confirm or discard this hypothesis.

We also endeavoured to compare our data with those produced by other serial crystallography approaches (SFX and Flow-SSX; see §[Sec sec2]2) on the same system (HEWL) and at the same temperature (RT). Table 3 ▶ shows that the three structures are roughly comparable in terms of *R*_split_ (0.158, 0.077 and 0.064 for the SFX, Flow-SSX and conservative nano data sets, respectively), 〈*I*/σ(*I*)〉 (7.4, 8.1 and 8.4, respectively), CC_1/2_ (not given for the SFX data set; 0.99 and 0.99 for the Flow-SSX and conservative nano data sets, respectively), resolution (1.9, 2.1 and 1.85 Å, respectively), *R*_work_ (0.196, 0.180 and 0.230, respectively) and *R*_free_ (0.229, 0.230 and 0.245, respectively). Yet, the dose and dose rate were 33 MGy and 8.3 × 10^14^ MGy s^−1^, respectively, for the SFX data set (Boutet *et al.*, 2012 ▶) and 0.3 MGy and 100 MGy s^−1^, respectively, for the Flow-SSX data set (Stellato *et al.*, 2014 ▶). Thus, the Flow-SSX data set is comparable to the nano data set in terms of dose rate (100 *versus* 291 MGy s^−1^) but not in terms of dose (0.3 *versus* 29.1 MGy). The SFX data set is comparable to the nano data set in terms of dose (33 *versus* 29.1 MG s^−1^) but not in terms of dose rate (8.3 × 10^14^ *versus* 291 MGy s^−1^). Nevertheless, radiation damage was clearly visible in both the *F*_o_^SFX^ − *F*_o_^nano^ and the *F*_o_^Flow-SSX^ − *F*_o_^nano^ Fourier difference maps (Fig. 5 ▶ *a*). This result was expected; the shortness of XFEL pulses indeed allows diffraction to occur before radiation damage can take place, whereas the reduced doses, together with a relatively high dose rate, have allowed mitigation of the appearance of specific damage in the Flow-SSX study. In conclusion, the three approaches yield data of comparable quality, suggesting that our raster-scanning approach could be a useful alternative to collect serial crystallography data from, for example, scarce crystalline samples. That our data sets are more radiation-damaged than those from SFX and Flow-SSX is acknowledged, yet this does not seem to significantly alter the quality of the structural information.

## 4. Discussion   

We have reported a new approach for the collection of high-resolution structural data from macromolecular crystals at RT using raster-scanning serial data collection. Two data sets were collected from tetragonal HEWL crystals on the ID13 beamline at the ESRF, using either a micro-focused (ID13-EH2) or a nano-focused (ID13-EH3) beam. Crystals were presented to the X-ray beam in an Si_3_N_4_ sandwich at RT (Fig. 1 ▶). During the whole experiment, including controls, 1 mg of crystalline sample was consumed.

In order to detect hits and correct for both distortion of the detector and background scattering (Fig. 3 ▶), we developed the Python-based software *NanoPeakCell* (Fig. 2 ▶) that performs these tasks in a timely (multi-processing) and user-friendly (graphical user interface) fashion. *NanoPeakCell* uses the FabIO library (Knudsen *et al.*, 2013 ▶) and is therefore able to process data from a variety of detector types including ADSC, MAR CCD and Pilatus detectors. It can also read HDF5, SACLA-HDF5 and LCLS-XTC files and can thus be used to process XFEL data (details to be published elsewhere). *NanoPeakCell* can output data in EDF (European Data Format), HDF5 (suitable for processing by *CrystFEL*) and/or ‘pickle’ (suitable for processing by *cctbx.xfel*) formats. In the latter case, the data are automatically padded so that the beam centre matches the centre of the image, as currently required for processing data with *cctbx.xfel*. *NanoPeakCell* can also generate input files for *CrystFEL* (geometry and beam description) and calculates a maximum projection of the corrected data on the fly (Supplementary Figs. S1*c* and S1*d*). Finally, *NanoPeakCell* can perform a Bragg-peak search on corrected images (Figs. 2 ▶ and 3 ▶); the resulting peak list is either included in a supplementary data set of the HDF5 file or printed in a text file. The *NanoPeakCell* peak list can be used as a guide for adjusting *CrystFEL* and/or *cctbx.xfel* spot-finding parameters, or directly fed into *CrystFEL* for indexing. In indexing tests performed using either the peak-finding algorithm implemented in *CrystFEL* or that implemented in *NanoPeakCell*, similar indexing rates were obtained (not shown). While peak searching is faster in *CrystFEL* than in *NanoPeakCell*, the computing cost is minor if *NanoPeakCell* is used to perform hit finding and data correction.

Indexing, integration and merging were performed using *CrystFEL* (Table 2 ▶). Good statistics were obtained for both the micro and the nano data sets, demonstrating that raster-scanning SSX using either micro- or nano-focused beams is a promising approach in RT macromolecular X-ray crystallo­graphy. The micro data set furthermore sustains that ∼6000 indexed hits are sufficient to obtain a reasonably good structure, at least when a molecular-replacement model is available (Figs. 4 ▶ *a* and 4 ▶ *c*, Table 2 ▶). Of note, background subtraction was compulsory to produce data sets of such quality.

As judged from statistical indicators such as 〈*I*/σ(*I*)〉, CC_1/2_ and *R*_split_ for the raw data, and *R*_free_ and *R*_work_ for the refined structures, the data obtained using raster-scanning SSX are of comparable quality to those produced by SFX and Flow-SSX (Table 3 ▶). The fairest comparison is between the Flow-SSX and the nano data sets as both were produced using a similar number of images (∼40 000 *versus* ∼46 000 indexed frames for the Flow-SSX and nano data sets, respectively) collected on a similar X-ray source. Differences between the two approaches are the crystal presentation procedure (crystal stream in Flow-SSX *versus* immobilized crystals on a solid support in the present study), the beam size (6 × 9 µm *versus* 0.15 × 0.175 µm), the dose per crystal (0.3 *versus* 29.1 MGy) and the dose rate (100 *versus* 291 MGy s^−1^). Of course, higher *R*_split_ and lower 〈*I*/σ(*I*)〉 values are observed in the highest resolution shells of the progressive nano data set (Tables 2 ▶ and 3 ▶), given that we intentionally applied less stringent resolution cutoffs in our progressive integrations. Indeed, we have relied on the observation of signal out to the edge of the detector in the maximum projections from indexed patterns (Supplementary Fig. S1) and on subsequently performed iterative pair refinement analyses (Supplementary Fig. 2 ▶) to determine resolution cutoffs for the progressive data sets. Statistics produced by the raster-scanning and the Flow-SSX approaches are nevertheless of similar quality when the high-resolution limit criteria are the same, *i.e.* 〈*I*/σ(*I*)〉 > 2 (1.9 and 2.37 for the Flow-SSX and conservative nano data sets, respectively) and *R*_split_ ≤ ∼50% (54.0 and 37.5% for the Flow-SSX and conservative nano data sets, respectively). Of note, the conservative nano data set extends to 1.85 Å resolution compared with 2.1 Å resolution for the Flow-SSX data set. Yet, our conservative nano data set suffers from a lower redundancy of 787 (430 in the highest resolution shell) compared with 1755 (1281) for the Flow-SSX data set. At least three factors could be contributing to this: the radius of the three-dimensional intensity profile used for the *CrystFEL* integration (5 × 10^−4^ Å^−1^ in the present study; not available for the Flow-SSX data set), the beam divergence (0.3 and 1.0 mrad, respectively) and the fact that in the flow approach the crystals do not remain still during exposure but sometimes roll, thus giving rise to pseudo-oscillation.

Regardless, it is clear that our data sets are more radiation-damaged than those produced by SFX (Boutet *et al.*, 2012 ▶) and Flow-SSX (Stellato *et al.*, 2014 ▶) (Fig. 5 ▶ *a*). Indeed, in an attempt to compensate for the lower flux and the relatively small irradiated volume at the nano-focused beamline (upper limit of 0.5 µm^3^), the nano data set was collected using a dose per image (29.1 MGy) just below the experimental dose limit defined by Garman and coworkers (30 MGy; Owen *et al.*, 2006 ▶) beyond which the cumulative information collected from a macromolecular crystal at 100 K is compromised. The micro data set was collected using a dose that was approximately ten times lower, *i.e.* 3.2 MGy. Given that radiation damage proceeds ∼100 times faster at RT than at 100 K (Nave & Garman, 2005 ▶), the theoretical estimate for the dose limit at RT is 0.3 MGy, implying that the micro and nano data sets are overdosed by factors of 10 and 100, respectively. Accordingly, specific damage was observed to disulfide bridges in the *F*_o_ − *F*_o_ maps calculated between our data sets and low-dose data sets collected either on a rotating anode at 100 K or *in situ* at RT (Fig. 5 ▶ *a*). Specific damage was also visible in *F*_o_ − *F*_o_ maps calculated between our data sets and either the SFX or the Flow-SSX data sets (Fig. 5 ▶ *a*).

Yet, no sign of specific damage was visible in the unbiased 2*mF*_o_ − *DF*_c_ and *mF*_o_ − *DF*_c_ maps calculated from both the micro and the nano data sets. More importantly, the structural information proved to be of sufficient quality to allow MR phasing with partial search models (down to 20% of the total atoms) and quasi-complete main-chain (98%) and side-chain (90–98%) fitting in the resulting maps of the HEWL sequence (Supplementary Table S1). A possible explanation for the limited damage observed in our data, and in the nano data set in particular, is that crystals suffer less damage when impinged with X-rays a single time (no cumulative effect) and at a very high dose rate (16 and 291 MGy s^−1^ for the micro and nano data sets, respectively). It has already been shown that data collection at high dose rates delays the appearance of radiation damage at RT (Owen *et al.*, 2014 ▶; Southworth-Davies *et al.*, 2007 ▶). Admittedly, the highest dose rate used in the study by Owen *et al.* (2014 ▶) was 25 MGy s^−1^ and the lag phase lasted about 500 kGy for all proteins, whereas in our experiments 500 kGy would have been delivered in 1.7 ms for the nano data set. Thus, the 500 kGy lag phase would have expired well within the exposure time for each image (0.1 s). It may thus be that for the magnitude-higher dose rate of the nano data set (291 MGy s^−1^) a further lag phase occurs. That the micro data set (16 MGy s^−1^) appears to be more damaged than the nano data set supports this hypothesis (Fig. 5 ▶ *a*). Again, additional experiments are required to confirm or discard this hypothesis.

A variety of sample-presentation strategies exist that either have been used or are amenable to SSX. These include flowing crystals across the X-ray beam by means of a capillary (Stellato *et al.*, 2014 ▶), a liquid jet (DePonte *et al.*, 2009 ▶; Sierra *et al.*, 2012 ▶) or a lipid cubic phase injector (Weierstall *et al.*, 2014 ▶) or ejecting them in droplets using acoustic waves (Soares *et al.*, 2011 ▶). Solid supports have also been used either to collect oscillation data (Zarrine-Afsar *et al.*, 2012 ▶) or still shots (Gebhardt *et al.*, 2014 ▶; Hunter *et al.*, 2014 ▶). Here, we used a Si_3_N_4_ membrane sandwich to present the crystals to the X-ray beam. This approach has advantages, including the small amount of micro-crystalline sample required (1 mg of protein compared with 250 mg in the study by Stellato *et al.*, 2014 ▶), an adjustable hit rate (∼10 and 40% in the micro and nano data sets, respectively), a high indexing rate (85 and 80% in the micro and nano data sets, respectively) and a minimized background scattering. Also, the proposed mounting procedure is virtually manipulation-free, suggesting that it could be used on fragile micro-crystals. Data collection at RT is another advantage, given the inherently flexible nature of biological material. Of course, this is a superiority to oscillation data collection at cryogenic temperature which raster-scanning SSX shares with the other serial crystallography approaches. It is tantalizing to propose that raster-scanning SSX could be utilized in kinetic crystallography experiments, in which a light source is used to ‘pump’ a macromolecular crystalline system (either a light-driven protein or a complex of a light-insensitive protein with a photocleavable caged compound) and the X-ray beam is used to ‘probe’ the resulting structure (Bourgeois & Royant, 2005 ▶; Colletier *et al.*, 2007 ▶). The attainable time resolution would essentially be limited by the length of the X-ray pulse and the detector readout, thus allowing time-resolved structural studies on the microsecond to second timescale, *i.e.* that pertinent to protein kinetics. X-rays could also be used as both the pump and the probe in the framework of ‘shoot-and-trap’ experiments (Colletier *et al.*, 2008 ▶) or to structurally monitor the appearance and evolution of radiation damage at RT in an oscillation-free, and thus damage-accumulation-free, fashion. The observation that the Cys64–Cys80 disulfide bridge is the most radiation-sensitive at RT (Fig. 5 ▶ *a*), instead of Cys30–Cys115 as at 100 K (Sutton *et al.*, 2013 ▶), advocates for the specific investigation of macromolecular radiation-sensitivity at RT.

A current weakness of SSX is the time that is required to collect a full data set. In the study by Stellato *et al.* (2014 ▶), 17 h were needed to produce a data set of 40 000 useable frames, owing to a comparatively low overall success rate. Although our success rates are higher overall, the nano data set consisting of 46 000 useable frames required 19 h to be produced owing to the comparatively slow readout time of our Frelon CCD detector. The ID13 beamline at the ESRF is now equipped with a new Dectris 4M Eiger detector, which will allow a drastic increase in the data-collection rate (1.3 ms sampling rate, 6 µs readout time). Also, Phase II of the ESRF upgrade, which involves a substantial upgrade of the storage ring, will lead to an increase of about a factor of 50 in brilliance. Based on this and other improvements, a 10 000-fold increase in the flux of the nano-beam can be expected, which will dramatically reduce the minimum exposure time required to collect high-resolution data.

## Supplementary Material

PDB reference: hen egg-white lysozyme, 4wg1

PDB reference: 4wg7

PDB reference: 4wl6

PDB reference: 4wl7

Supplementary table and figures. DOI: 10.1107/S1399004715004514/kw5115sup1.pdf

## Figures and Tables

**Table 1:** Data-collection parameters

Parameter	Micro	Nano
Beam size (vertical horizontal) (FWHM) (m)	1.5 2.5	0.150 0.180
Beam divergence (mrad)	1.0	0.3
Wavelength ()	0.954	0.832
Beam fluence (photonss^1^)	1.00 10^11^	1.70 10^10^
Flux density (photonss^1^mm^2^)	2.67 10^16^	6.48 10^17^
Brilliance (photonss^1^mm^2^mrad^2^)	2.67 10^22^	6.09 10^24^
Bandwidth ()	1.00 10^4^	1.00 10^4^
Exposure time per frame (s)	0.2	0.1
Dose per crystal† (MGy)	3.2	29.1
Dose rate (MGys^1^)	16	291

† As calculated using *RADDOSE* (Paithankar *et al.*, 2009 ▶).

**Table 2:** Crystallographic refinement statistics Values in parentheses are for the highest resolution shell.

Name of data set	Conservative micro	Conservative nano	Conservative reduced nano	Progressive micro	Progressive nano	Progressive reduced nano
Space group	*P*4_3_2_1_2					
Unit-cell parameters						
*a* = *b* ()	78.0 0.3	78.0 0.2	78.0 0.2	78.0 0.2	78.0 0.2	78.0 0.2
*c* ()	38.3 0.3	38.5 0.2	38.5 0.2	38.3 0.3	38.5 0.2	38.5 0.2
= = ()	90	90	90	90	90	90
No. of collected images	69319	139985	n/a	69319	139985	n/a
No. of hits	6862	58647	n/a	6862	58647	n/a
Resolution ()	501.95 (2.051.95)	501.85 (1.951.85)	501.95 (2.051.95)	501.70 (1.781.70)	501.70 (1.781.70)	501.70 (1.781.70)
No. of indexed images	5966	46516	5966	5966	46516	5966
No. of reflections	1448031	9069707	1036661	1594657	10710039	1371898
No. of unique reflections	9091	10640	9132	13543	13608	13606
Completeness (%)	100 (100)	100 (100)	100 (100)	100 (100)	100 (100)	100 (100)
Average multiplicity	159 (70)	852 (644)	114 (86)	115 (18)	787 (430)	101 (61)
*I*/(*I*)	4.5 (2.4)	8.4 (2.4)	3.5 (1.5)	3.5 (0.6)	6.8 (0.9)	2.5 (0.3)
*R*_split_ intensity agreement of semi-data sets†	0.172 (0.390)	0.064 (0.375)	0.200 (0.586)	0.178 (1.339)	0.066 (1.155)	0.208 (3.352)
*R*_merge_(*I*)	0.238 (0.541)	0.090 (0.523)	0.275 (0.800)	0.247 (1.743)	0.093 (1.697)	0.286 (4.643)
*R*_merge_(*F*)	0.146 (0.280)	0.067 (0.221)	0.165 (0.316)	0.163 (0.486)	0.074 (0.387)	0.177 (0.440)
CC_1/2_ correlation of semi-data sets‡	0.99 (0.94)	0.99 (0.98)	0.98 (0.94)	0.99 (0.66)	0.99 (0.85)	0.99 (0.50)
Wilson *B* § (^2^)	32	38	35	34	42	42
*R*_free_	0.264	0.245	0.261	0.255	0.239	0.264
*R*_work_	0.214	0.230	0.203	0.226	0.204	0.223
R.m.s. deviations from ideal values¶						
Bonds ()	0.006	0.006	0.005	0.006	0.006	0.005
Angles ()	0.872	0.901	0.929	0.864	0.935	0.878
Average *B* value (^2^)	39.9	62.3	44.0	40.8	44.20	45.20
Clashscore††	6.31	4.23	3.11	5.34	4.23	5.17
Ramachandran plot††						
Most favoured	98.5	98.5	97.0	97.7	98.5	97.8
Allowed	1.5	1.5	3.0	2.3	1.5	2.2
Disallowed	0	0	0	0	0	0
Rotamer outliers††	0	2.7	2.7	0	1.8	3.5
PDB entry	4wl7	4wl6	n/a	4wg1	4wg7	n/a

† As defined in White *et al.* (2012 ▶).

‡ As defined in Karplus Diederichs (2012 ▶).

§ As calculated by *TRUNCATE* (French Wilson, 1978 ▶) in *CCP*4 (Winn *et al.*, 2011 ▶).

¶ As defined in Engh Huber (1991 ▶).

†† As calculated by *MolProbity* (Chen *et al.*, 2010 ▶).

**Table 3:** Comparison of *CrystFEL* indexing statistics for data collected from lysozyme microcrystals using SFX (Boutet *et al.*, 2012 ▶), Flow-SSX (Stellato *et al.*, 2014 ▶) and raster-scanning SSX (this work) Values in parentheses are for the highest resolution shell.

Type of experiment	SFX, 40fs pulses (Boutet *et al.*, 2012 ▶)	SFX, 5fs pulses (Boutet *et al.*, 2012 ▶)	Flow-SSX (Stellato *et al.*, 2014 ▶)	Raster-scanning SSX, conservative micro	Raster-scanning SSX, conservative nano
PDB entry	4et8	4et9	4o34	4wg1	4wg7
Dose per crystal† (MGy)	33	2.9	0.3	3.2	29.1
Dose rate (MGys^1^)	8.3 10^14^	5.8 10^14^	100	16	291
Unit-cell parameters ()					
*a*	79.0	79.0	79.5 0.3	78.0 0.3	78.0 0.2
*b*	79.0	79.0	79.4 0.2	78.0 0.3	78.0 0.2
*c*	38.0	38.0	38.4 0.2	38.3 0.3	38.5 0.2
Upper limit for irradiated volume‡ (m^3^)	3	3	125	75	0.5
Resolution ()	35.901.90 (2.001.90)	35.901.90 (2.001.90)	39.652.10 (n/a)	501.95 (2.051.95)	501.85 (1.951.85)
*I*/(*I*)	7.4 (2.8)	7.3 (3.1)	8.1 (1.9)	4.5 (2.4)	8.4 (2.4)
*R*_split_ §	0.158 (n/a)	0.159 (n/a)	0.077 (0.540)	0.172 (0.390)	0.064 (0.375)
*R*_merge_(*I*)	n/a	n/a	n/a	0.238 (0.541)	0.090 (0.523)
*R*_merge_(*F*)	n/a	n/a	n/a	0.146 (0.280)	0.067 (0.221)
CC_1/2_ [Table-fn tfn10]	n/a	n/a	0.99 (0.90)	0.99 (0.94)	0.99 (0.98)
Wilson *B* [Table-fn tfn11] (^2^)	28	29	44	32	38
*R*_free_	0.229	0.227	0.230	0.264	0.245
*R*_work_	0.196	0.189	0.180	0.214	0.230
R.m.s. deviations from ideal values					
Bond lengths ()	0.006	0.006	0.007	0.006	0.006
Bond lengths ()	1.000	1.030	1.080	0.872	0.901
No. of frames collected	1.47 10^6^	1.99 10^6^	1.50 10^6^	0.07 10^6^	0.14 10^6^
Hit rate (%)	4.5	2.0	11.2	9.9	41.9
No. of indexed frames	12247	10575	40233	5966	35446
Indexing rate (%)	18.4	26.4	24.0	86.9	79.3
Overall indexing rate (%)	0.83	0.53	2.67	8.60	33.23

† As calculated using *RADDOSE* (Paithankar *et al.*, 2009 ▶).

‡ Either the beam surface multiplied by the largest dimension of the crystals (if the beam is smaller than the crystals) or the crystal volume (if the beam is larger than the crystals).

§ As defined in White *et al.* (2012 ▶).

¶ As defined in Karplus Diederichs (2012 ▶).

†† As calculated by *TRUNCATE* (French Wilson, 1978 ▶) in *CCP*4 (Winn *et al.*, 2011 ▶).
