# Supplementary material for: Raster-scanning serial protein crystallography using micro- and nano-focused synchrotron beams
Source: Acta Crystallogr D Biol Crystallogr. 2015 Apr 25;71(Pt 5):1184–96. doi: 10.1107/S1399004715004514 (PMC4427202; doi:10.1107/S1399004715004514)
Supplement: Supplementary file 1 [file d-71-01184-sup1.pdf]

BIOLOGICAL CRYSTALLOGRAPHY

Volume 71 (2015)

Supporting information for article:

Raster-scanning serial protein crystallography using micro and nano-focused synchrotron beams

Nicolas Coquelle, Aaron S. Brewster, Ulrike Kapp, Shilova Anastasya, Britta Weinhausen, Manfred Burghammer and Jacques-Philippe Colletier

Supplementary Figure 1: Overall quality and consistency of the progressive "micro" and "nano" dataset. (A, B) Signal to noise ratio as a function of resolution in the progressive "micro" (A) and "nano" datasets (B), respectively. (C, D) Maximum projection of the patterns used to generate the "micro" (C) and "nano" datasets (D), respectively; for both, the right inset shows a zoom at high resolution. The detector resolution is 2.2/1.6 Å at the edge/corner for the "micro" (C), and 1.75/1.3 Å at the edge/corner for the "nano" (D). (E) 1D radial intensity profile of the "micro" maximum projection (C), integrated azimuthally with Fit2D (Hammersley *et al.*, 1996) over 360° and plotted as a function of 2theta (lower axis) and the resolution (upper axis); the inset shows a zoom in the 2.0-1.6 Å resolution range. (F) 1D radial intensity profile of the "nano" maximum projection (D), integrated azimuthally with Fit2D over 360° and plotted as a function of 2theta (lower axis) and the resolution (upper axis); the inset shows a zoom in the 2.0-1.3 Å resolution range.

Supplementary Figure 2: Determination of effective high-resolution cut-offs for the "micro" and "nano" dataset. (A-D) Heat-maps of R_{split} (A, B) and $CC^{1/2}$ (C, D) in the "micro" (A, C) and nano (B, D) datasets, respectively. These plots illustrate the convergence of our integrations. (E, F) Iterative-pair refinement was performed to validate the 1.7 Å resolution cut-offs of the progressive "micro" (E) and "nano" (F) datasets. In brief, the final model was refined at various resolutions ranging from 2.2 to 1.7 Å, with incremental steps of 0.1 Å. For each increment from resolution A to B, the difference in R-work and R-free values computed at resolution A, for models refined at resolution A and B; the differences in R-work and Rfree were plotted.

Supplementary Figure 3: Overall R_{iso} between various HEWL datasets. Unweighted and variance-weighted R_{iso} are shown in the lower left and upper right halves of the heat-map, respectively. Data were scaled using SCALEIT (Bailey et al., 1994).

Supplementary Figure 4: R_{iso} between various HEWL datasets plotted as a function of resolution. Unweighted and variance-weighted R_{iso} are shown in the blue left and red, respectively. Data were scaled using SCALEIT (Bailey et al., 1994).

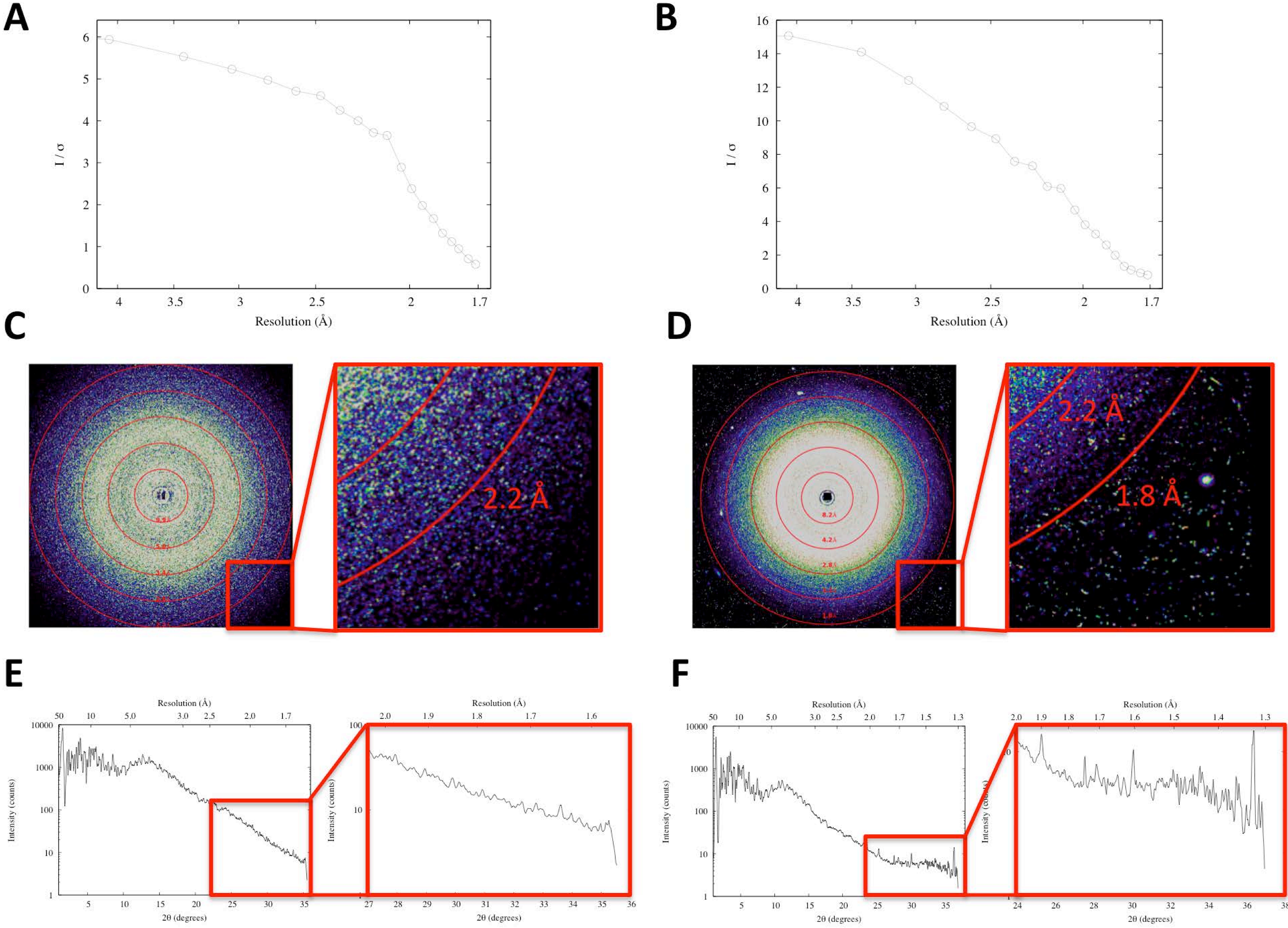

A
B
C
D
E
F
I / σ
Resolution (Å)
4
3.5
3
2.5
2
1.7
0
1
2
3
4
5
6
8
10
12
14
16
2.2 Å
1.8 Å
Resolution (Å)
50
10
5.0
3.0
2.5
2.0
1.9
1.8
1.7
1.6
1.5
1.4
1.3
Intensity (counts)
10000
1000
100
10
1
2θ (degrees)

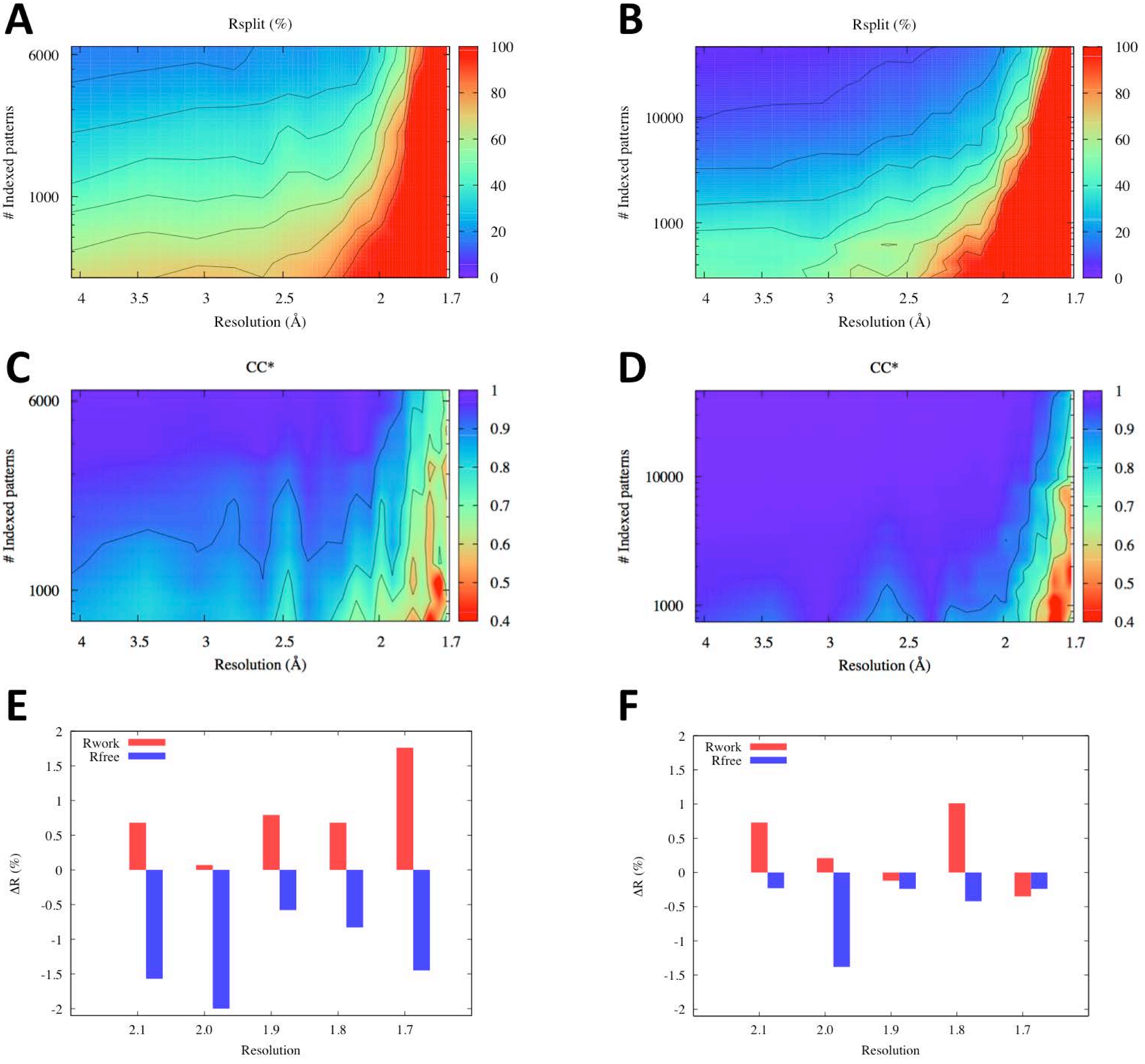
A
Rsplit (%)
Indexed patterns
Resolution (Å)
B
Rsplit (%)
Indexed patterns
Resolution (Å)
C
CC*
Indexed patterns
Resolution (Å)
D
CC*
Indexed patterns
Resolution (Å)
E
Rwork
Rfree
ΔR (%)
Resolution
F
Rwork
Rfree
ΔR (%)
Resolution

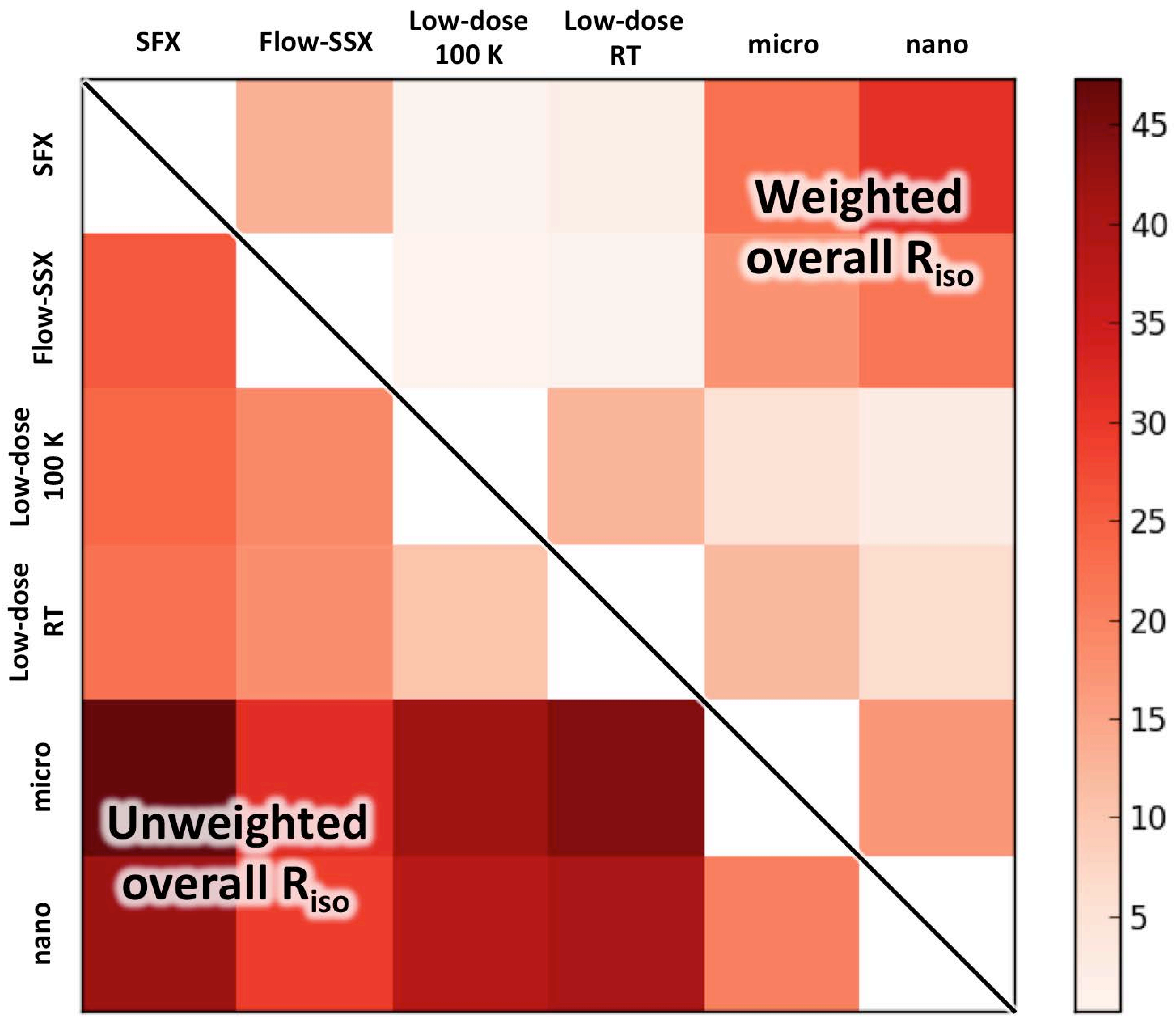
SFX
Flow-SSX
Low-dose 100 K
Low-dose RT
micro
nano
SFX
Flow-SSX
Low-dose 100 K
Low-dose RT
micro
nano
Weighted overall R_{iso}
Unweighted overall R_{iso}
45
40
35
30
25
20
15
10
5

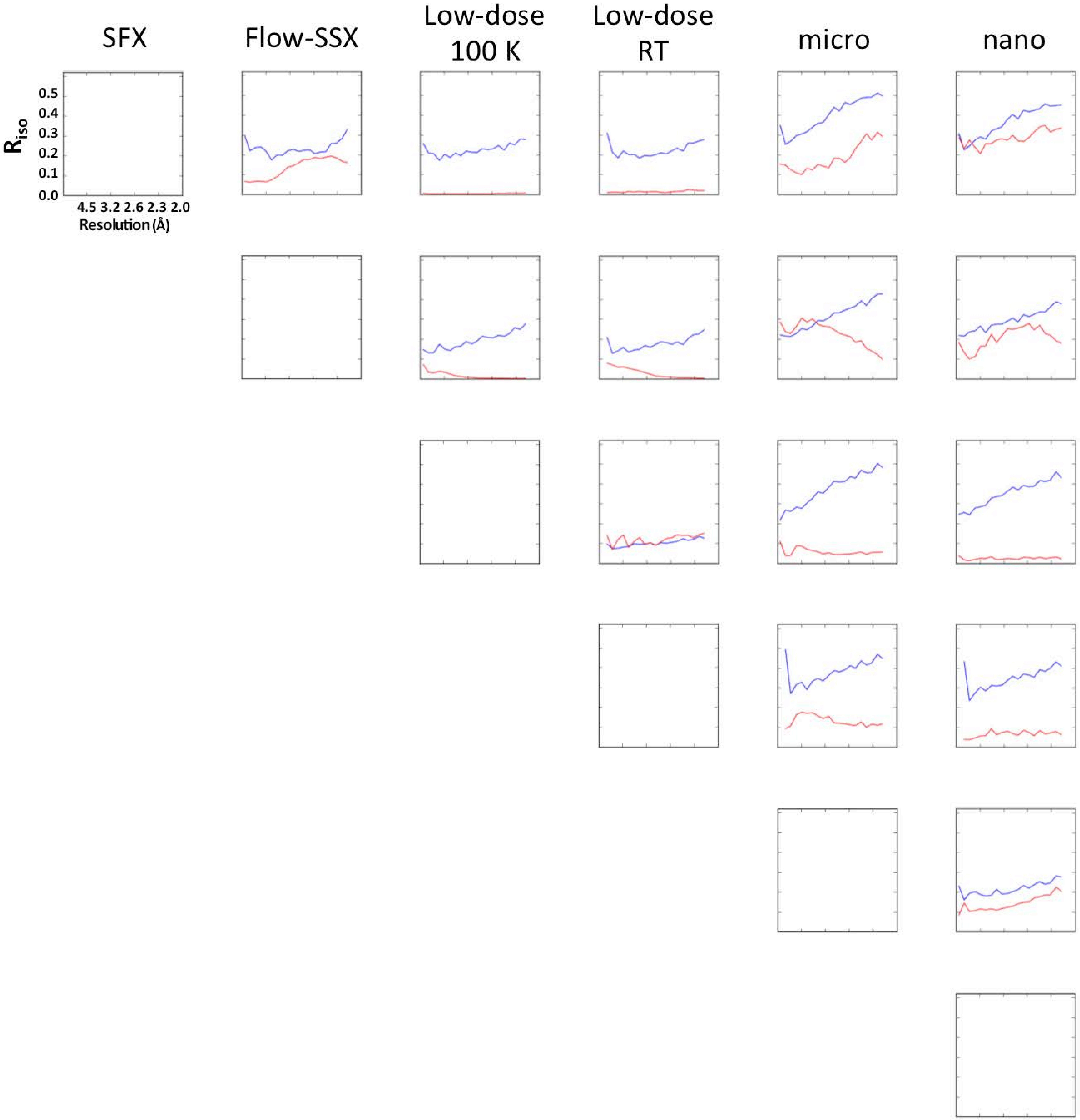

SFX
Flow-SSX
Low-dose 100 K
Low-dose RT
micro
nano
R_iso
0.5
0.4
0.3
0.2
0.1
0.0
4.5 3.2 2.6 2.3 2.0
Resolution (Å)

Supplementary Table 1: Automated fitting of the HEWL sequence into experimental maps obtained by MR phasing of the "progressive nano" dataset with various starting models.

	Main and side chain atoms of residues 1-127	Main and side chain atoms of residues 1-65	Main and side chain atoms of residues 66-127	Main chain atoms of residues 1-127	Main chain atoms of residues 1-65	Main chain atoms of residues 65-127
**Molecular Replacement model**	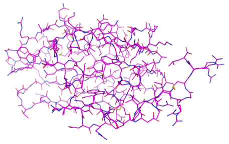	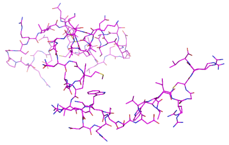	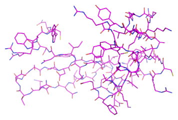	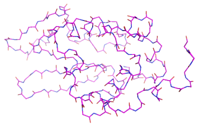	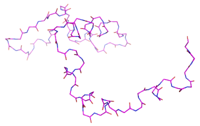	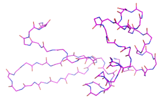
**No. of mainchain / sidechain (of total 129)**	**126 / 126**	**117 / 117**	**125 / 125**	**125 / 125**	**127 / 117**	**126 / 126**
**R_{free} / R_{work}**	**0.29 / 0.24**	**0.30 / 0.28**	**0.31 / 0.26**	**0.30 / 0.26**	**0.29 / 0.25**	**0.29 / 0.25**
**Map-to-model correlation coefficient**	**0.82**	**0.80**	**0.80**	**0.82**	**0.81**	**0.82**
